# Modified tibial plateau levelling osteotomy to treat lateral patellar luxation and cranial cruciate ligament deficiency in a dog

**DOI:** 10.1002/ccr3.4365

**Published:** 2021-08-16

**Authors:** Eloy H. P. Curuci, Filippo J. L. Bernardes, Bruno W. Minto

**Affiliations:** ^1^ Faculty of Agrarian and Veterinary Sciences Department of Veterinary Clinic and Surgery São Paulo State University Jaboticabal Brazil

**Keywords:** canid, orthopaedics, surgery, veterinary

## Abstract

The use of modified tibial plateau leveling osteotomy adapted for correction of the lateral patellar dislocation was effective, allowing simultaneous treatment of lateral patellar luxation and cranial cruciate ligament deficiency.

## Graphical abstract

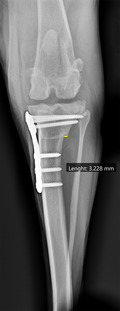

## 1 INTRODUCTION

A 4‐year‐old female Boxer dog weighing 36.6 kg, previously diagnosed with a grade II lateral patellar luxation, was presented with cruciate ligament rupture. The patient underwent lateral modified tibial plateau leveling osteotomy with partial meniscectomy and *en bloc* trochleoplasty. The technique was effective to concurrently treat both conditions.

Cranial cruciate ligament (CCL) insufficiency is one of the most common orthopedic disease surgicaly treated in dogs.^1^ Patellar luxation (PL) also occurs with high prevalence.^1^, ^2^ The two stifle pathologies often occur concurrently in dogs, with between 15.7% and 25% of dogs having both diseases. ^3^, ^4^

Tibial plateau leveling osteotomy (TPLO), originally described by Slocum and Slocum,^5^ promotes dynamic stabilization of the stifle by reducing the inclination of the tibial plateau, preventing tibial cranial translation. Several modifications and adaptations have improved the effectiveness of the technique.^6^ It has been used successfully for the treatment of CCL deficiency in dogs for the past three decades. A combined surgical technique that addresses PL and CCL deficiency in a single procedure allows both conditions to be treated simultaneously with a reduction in surgical trauma and recovery.^7^

The tibial tuberosity transposition (TTT) technique has been used for the treatment of PL for more than 60 years^8^ and it has recently been combined with TPLO in an attempt to treat both conditions simultaneously.^7^, ^9^ The modified TPLO proposed by Langenbach and Marcellin‐Little ^9^ involves lateral translation and external rotation of the tibial tuberosity in relation to the osteotomized fragment, to mimic the TTT for cases of medial dislocation of the patella. Inspired by this technique, and with the immediate need to simultaneously manage lateral dislocation of the patella and achieve dynamic stabilization of the stifle due to CCL deficiency, we report the use of an adaptation of modified TPLO to correct both conditions.

## 2 CASE REPORT

A 4‐year‐old, 36.6 kg, female Boxer dog, diagnosed 2 years previously with lateral patellar luxation (LPL), was presented with acute left hindlimb lameness. Orthopedic examination revealed a typical CCL deficiency, cranial displacement of the tibia and positive cranial drawer and tibial compression tests. LPL and laxity of the left hip joint were also observed.

**FIGURE 1:**
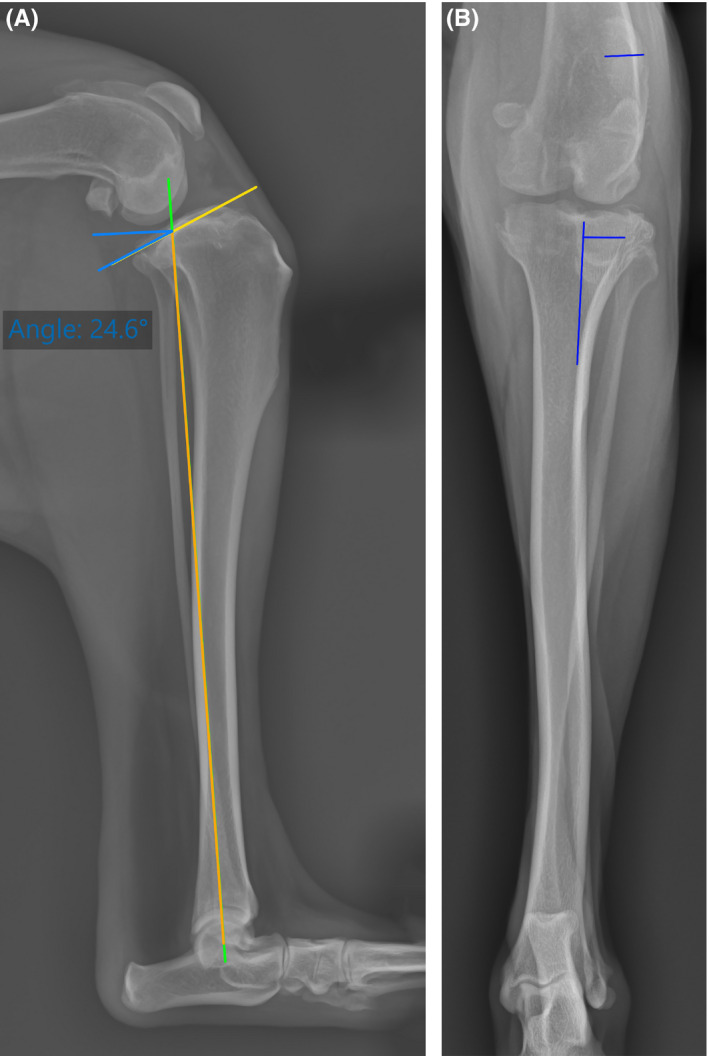
Mediolateral A, and craniocaudal B, preoperative radiographs of the left stifle with tibial tuberosity external torsion leading to a grade II lateral patellar luxation and tibial thrust as a result of cranial cruciate ligament rupture. A, Measurement of preoperative tibial plateau angle of 24.6°. B, Patella and tibial tuberosity misalignment

The patient was premedicated with acepromazine (0.03 mg/kg IM) and pethidine (3 mg/kg IM), and anesthesia was induced with propofol (4 mg/kg IV) and maintained with isoflurane. Intravenous cefazolin (30 mg/kg IV) was administered 30 minutes before surgery. Epidural analgesia was administered using ropivacaine (1 mg/kg) in combination with methadone (0.1 mg/kg). Mediolateral radiographs (centered at the stifle) were taken with the stifle and hock held at 90° (Figure 1A). Craniocaudal projections included hock and stifle with the beam centered on the stifle (Figure 1B).

The preoperative radiographic evaluation showed patellar lateralization from the trochlear groove. A 3.2 mm tibial tuberosity internal torsion was measured on a craniocaudal radiograph, using the proximal and distal tibial angle measurement described by Dismukes et al^10^ The tibial plateau angle (TPA) was measured on preoperative (24°) and postoperative (5°) radiographs using the method proposed by Slocum and Devine.^11^ The orientation of the femoral trochlea in relation to the distal portion of the femoral shaft as seen on craniocaudal radiographs was measured.

A craniomedial approach to the stifle was performed. Rupture of the CCL and LPL were confirmed. Remnants of the CCL and the caudal portion of the injured medial meniscus were removed. A recession wedge trochleoplasty was performed. The TPLO procedure was performed following the standard Slocum technique with a 27 mm saw blade.^5^ The jig was not removed after completion of the curved cut. The tibial plateau was leveled by an 8.9 mm rotation and simultaneously aligned with the quadriceps mechanism by medially translating the distal tibial fragment, reaching a 3.22 mm postoperative translation. At this point the distal part of the jig was released to allow the medial displacement of the distal tibial fragment in relation to the osteotomized proximal fragment, resulting in a medial transposition of the tibial tuberosity and realignment of the mechanical axis of the pelvic limb.

The tibial plateau was temporarily stabilized with a 2 mm Steinmann pin and the patella was checked for stability. A Fixin (Intrauma S.p.A) 3.5 mm TPLO Clover Large plate and six screws were used. The plate did not need any additional contouring to fit the medial surface of the proximal tibia. It was pre‐fixed in place with two K‐wire through small holes designed for this purpose. A conventional screw was inserted, without tightening, until it touched the surface of the plate. The proximal fragment was secured using three locking screws, taking care to maintain the correct position of the plate and direction of the screws. The conventional screw was then tightened to compress the osteotomy line. Finally, the two remaining distal locking screws were inserted.

The stifle was re‐checked for patella stability, quadriceps alignment, and absence of cranial tibial thrust. The stifle was individually closed in layers (synovial membrane, fascia and subcutaneous tissues) using 3‐0 monofilament absorbable poliglecaprone 25 and non‐absorbable 3‐0 nylon sutures in the skin. The postoperative radiographs were acquired followed by placement of a modified Robert Jones bandage.

## 3 RESULTS

**FIGURE 2:**
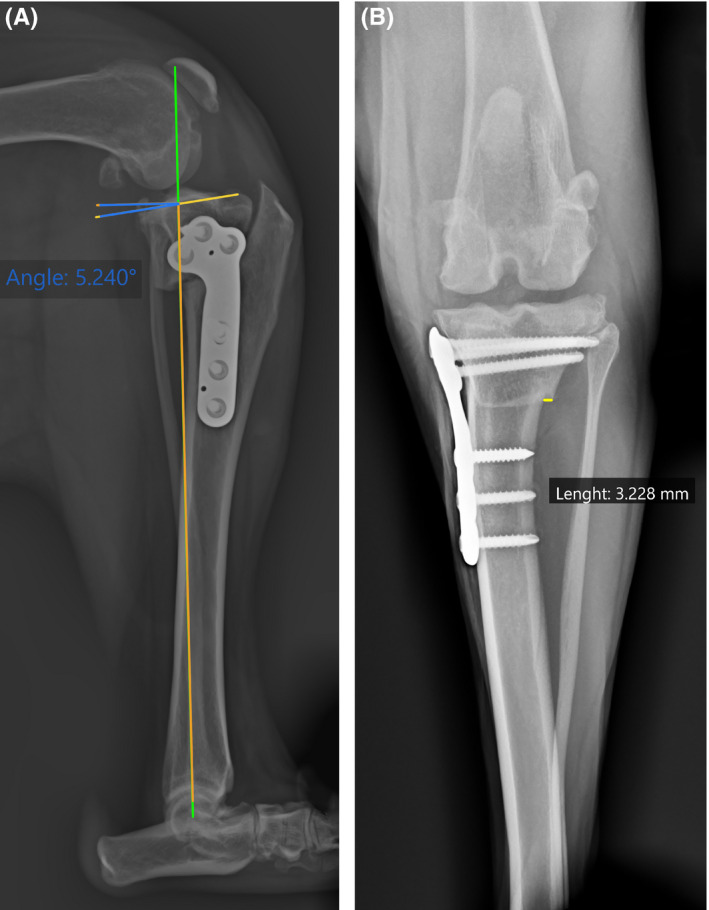
Mediolateral A, and craniocaudal B, left stifle radiographs taken immediately after a lateral modified tibial plateau leveling osteotomy with a 3.5 mm tibial plateau leveling osteotomy (TPLO) plate. A, Postoperative final tibial plateau angle (TPA) of 5.2 and proximal tibia alignment. B, A 3.22 mm lateral overhang created after the translation

**FIGURE 3:**
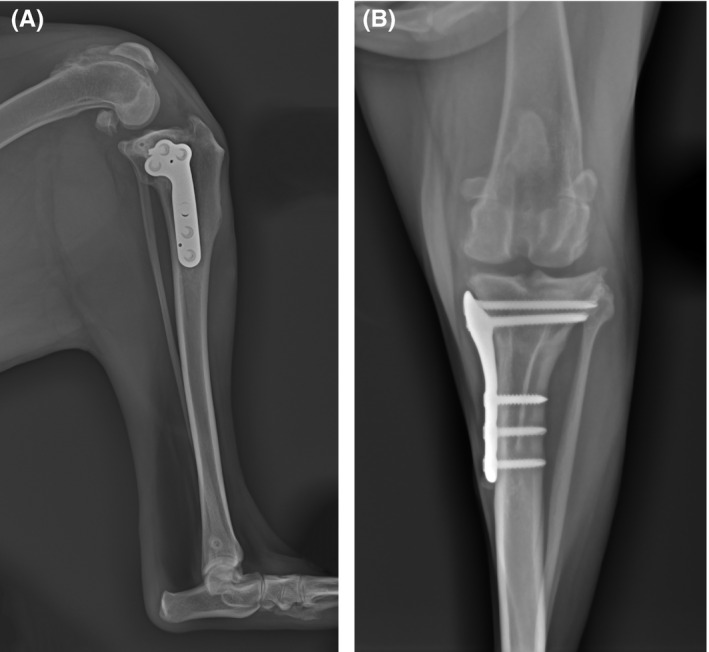
Mediolateral A, and craniocaudal B, left stifle radiographs taken 8 weeks after a lateral modified tibial plateau leveling osteotomy with a 3.5 mm tibial plateau levelling osteotomy (TPLO) plate. A, Osteotomy line is not apparent, showing bone union. B, Patella and tibial tuberosity alignment

Immediate postoperative radiographs revealed correction of tibial internal torsion, with a 5° final TPA (Figure 2A) and medial translation of the distal tibial fragment (Figure 2B). Physical examination, subjective gait analysis of the limb function, and radiographic assessment were performed at 4, 8, and 12 weeks postoperatively. The dog was weight bearing in the first week, recovered full limb function with no signs of relaxation or lameness by 4 weeks. Satisfactory radiographic healing of the osteotomy line was observed at 8 weeks postoperatively (Figure 3). No complications were reported nor additional procedures needed.

## 4 DISCUSSION

For patients with concurrent LPL and CCL deficiency management of both conditions is necessary to achieve a normal function of the stifle joint. Although a single procedure is preferential, to minimize surgical morbidity and improve the result, in some cases a two‐staged procedure is used.^9^ There are a number of techniques to manage LPL and CCL deficiency in a single procedure, such as the modified triple tibial osteotomy (mTTO), tibial tuberosity transposition advancement (TTTA), or combined TPLO and TTT, these have different grades of technical difficulty and complication rates.^7^, ^12^, ^13^

Inspired by the concepts of the modified TPLO for concurrent treatment of medial patellar luxation (MPL) as previously described by Langenbach & Marcellin‐Little^9^ and Flesher et al^14^ and considering the better outcomes with simultaneous treatment of both conditions, we performed in this case a lateral modified TPLO (LmodTPLO) for simultaneous management of LPL and CCL deficiency. An excellent clinical outcome was achieved, with no technical difficulties in performing this technique. Although this is only a single case report, we believe that this technique can be standardized to treat selected patients with both orthopedic conditions.

Langenbach & Marcellin‐Little^9^ and Flesher et al^14^ describe a TPLO modification to treat concurrent CCL disease and MPL, laterally translating and externally rotating the principal tibial portion. This translation creates an overhang on the medial aspect of the tibia. The modified TPLO mimics the TTT function, a technique in which the tibial tuberosity is osteotomized and laterally, or medially, translated to the contralateral side to the patellar luxation, and stabilized with a figure‐of‐eight tension band or other implant.^8^ Our procedure was based on this technique for MPL, the translation was performed to the opposite side to treat the LPL, realigning the quadriceps extensor mechanism and producing dynamic stabilization of the stifle, neutralizing the tibial thrust.

The tibial translation was performed using two different methods described in the previous published papers reporting modified TPLO. In the first modified TPLO technique the distal tibial fragment was laterally translated by 3‐6 mm, externally and abaxially rotated,9 whilst in Flesher et al14 the proximal tibial segment was medially translated 1‐5 mm. The technique we report is similar to that of Flesher et al14 however, instead of relocating the proximal segment the distal tibial segment was translated. In contrast to the previous reports of MPL, in our study a LPL was present.

The Slocum jig was used during TPLO to produce correct alignment during the osteosynthesis as described in the original TPLO technique.^5^ Even though it has been shown that the jig is not essential to perform a TPLO, we used the jig to maintain the alignment during the translation.^15^ It was necessary to open the distal arm of the jig to perform the medial translation of the distal tibial segment, using the jig as a guide made the translation more accurate.

In the modified TPLO for MPL locking implants are essential to provide satisfactory stabilization, since the bone‐plate contact is limited on the proximal osteotomized tibial fragment. Since the LmodTPLO involves tibial translation only in a single transverse plane it produces greater tibial tuberosity stability than other modified TPLO procedures and no additional osteotomies are necessary. This reduces the risk of complications such as tibial tuberosity fracture, patella baja or patella alta that can occur with TTTA or other techniques.^13^ Additionally, fewer metallic implants are used, reducing the risk of implant migration, irritation, or long‐term infection from K‐wires or Steinmann pins in a figure‐of‐eight tension band.^7^

When the modified TPLO is performed to treat MPL, the plate must be contoured due to the proximal fragment medialization.^9^, ^14^ In contrast, no plate contouring was required in our case since the overhang of the proximal fragment was decreased as a result of medial shaft translation. However, there is no reference standard when deciding whether or not to contour the plate in TPLOm lateral procedures and surgeons will face different decisions on a case‐by‐case basis. Contouring decisions will depend on individual anatomical features and the magnitude of the translation needed.

The evaluation of this technique and its outcome is severely limited by the absence of postoperative force‐plate gait analysis and the report of only one case. Further studies with long‐term follow‐up and larger case numbers are needed to evaluate the viability and repeatability of the technique. To date, there are no previous reports of modified TPLO for LPL (LmodTPLO) in dogs.

Medial translation of distal tibial portion was effective for the management of concurrent CCL deficiency and LPL in a dog. The procedure requires a single osteotomy resulting in an excellent clinical outcome and satisfactory bone healing.

## CONFLICT OF INTEREST

None declared.

## AUTHOR CONTRIBUTIONS

EHPC, FJLB, and BWM: contributed to conception of study, study design, acquisition of data and data analysis and interpretation. All authors drafted, revised and approved the submitted manuscript.

## ETHICAL APPROVAL

Written informed consent was obtained from the animal owner for publication of this case report and the accompanying images.

## Data Availability

All relevant clinical data and images are included in this report. Any additional information is available from the author following reasonable request.
